# The Impact of Nicotine Usage Across Body Contouring Surgery Outcomes and Opioid Usage

**DOI:** 10.1007/s00266-026-06033-5

**Published:** 2026-07-29

**Authors:** Angad S. Sidhu, Avani A. Chopra, Daniel J. Konig, George S. Corpuz, Cynthia Sung, Aanya Chopra, Cristiane M. Ueno, Mary E. Lester, Ivan Hadad

**Affiliations:** 1 Division of Plastic and Reconstructive Surgery, Indiana University School of Medicine, 545 Barnhill Drive, Emerson Hall 232, Indianapolis, IN 46202 USA; 2 Penn State College of Medicine, Hershey, PA USA

**Keywords:** Aesthetic surgery, Plastic surgery, Nicotine, Tobacco, Body contouring, Opioids

## Abstract

**Background:**

Nicotine use is a well-established risk factor for surgical wound complications and overall postoperative morbidity;however, its impact on elective aesthetic procedures such as body contouring surgery remains poorly characterized.

**Methods:**

Using a large de-identifi ed electronic health record database (TriNetX), adult patients who underwent bodycontouring procedures between 2005 and 2025 were identifi ed and propensity score matched based on history ofnicotine dependence. Postoperative outcomes at 90-days were compared between patients with and withoutnicotine dependence, including wound complications, complications requiring operative intervention, seroma,venous thromboembolism, and opioid prescriptions.

**Results:**

A total of 110,287 patients were identifi ed. After matching, each cohort had 17,065 patients. At 90-dayspostoperatively, patients with nicotine dependence demonstrated signifi cantly higher odds of wound complications,wound complications requiring operative intervention, seroma formation, and opioid prescriptions compared to non-nicotine users. No signifi cant diff erences were observed in venous thromboembolism rates between groups.

**Conclusion:**

These fi ndings suggest that nicotine dependence is associated with increased postoperative morbidity followingbody contouring procedures, particularly with respect to wound-related complications and opioid utilization. Routinepreoperative nicotine screening, patient education, and cessation interventions may play an important role inmitigating postoperative complications and improving outcomes in patients undergoing elective aesthetic bodycontouring surgery

*Level of Evidence III* This journal requires that authors assign a level of evidence to each article. For a full description of these Evidence-Based Medicine ratings, please refer to the Table of Contents or the online Instructions to Authors www.springer.com/00266.

**Supplementary Information:**

The online version contains supplementary material available at https://doi.org/10.1007/s00266-026-06033-5.

## Introduction

Nicotine usage, such as cigarette smoking, chewing tobacco, or vaping, is a common, modifiable perioperative risk factor that has been associated with increased wound healing complications through its vasoconstrictive effects [1, 2]. Body contouring surgeries, such as abdominoplasty, liposuction, and body-lifts, require extensive tissue plane dissection and rely on adequate microvascular perfusion for proper healing, making them susceptible to nicotine-related morbidity [3]. Unlike reconstructive procedures, the indications for body contouring are driven by patient preference in addition to medical necessity, placing greater importance on accurate preoperative risk stratification and evidence-based counseling to optimize outcomes and patient satisfaction. Beyond wound complications, nicotine use may also affect postoperative pain control and recovery, making prescription opioid fills a clinically relevant outcome in patients undergoing aesthetic procedures [4]. This study sought to examine nicotine dependence on postoperative outcomes following elective body contouring procedures.

## Methods

The TriNetX (TriNetX LLC, Cambridge, MA), a global federated health research network providing access to geographically aggregated and de-identified electronic medical records from large Healthcare Organizations, was queried (2005-2025) to identify patients ≥18 who underwent body contouring procedures (Supplemental Table 1). Two cohorts were created with Group 1 consisting of adults with a diagnosed history of nicotine dependence 30 days prior to surgery (ICD-10: F17 and Z87.891) and Group 2 having had no history of nicotine dependence. Propensity score matching was performed using age, sex, race, and the modified Charlson Comorbidity Index (Table 1). Standardized mean differences were used to evaluate balancing of cohorts. Outcomes of interest included wound complications, wound healing complications (wound debridement and drainage), seroma, venous thromboembolism, and opioid prescription events at 90-days postoperatively. Odds ratios with a 95% confidence interval and risk percentages were calculated on the TriNetX platform with p values <0.05 considered statistically significant.

**Table 1:** Propensity-matched patient demographics

Characteristics	Cohort 1	Cohort 2	SMD*
Mean age, years	49.9 ± 14.2	50 ± 14.3	0.011
Male, n (%)	1163 (6.8)	1060 (6.2)	0.024
Female, n (%)	15872 (93.0)	15974 (93.6)	0.024
Acute myocardial infarction, n (%)	70 (0.4)	57 (0.3)	0.013
Cancer, n (%)	6945 (40.7)	7027 (41.2)	0.010
Cerebral vascular accident, n (%)	102 (0.6)	85 (0.5)	0.013
Congestive heart failure, n (%)	310 (1.8)	248 (1.5)	0.029
Connective tissue disorder, n (%)	238 (1.4)	252 (1.5)	0.007
Dementia, n (%)	18 (0.1)	14 (0.1)	0.008
Diabetes mellitus, n (%)	1858 (10.9)	1917 (11.2)	0.011
Hemiplegia, n (%)	17 (0.1)	12 (0.1)	0.010
HIV, n (%)	71 (0.4)	63 (0.4)	0.007
Liver disease, n (%)	810 (4.7)	813 (4.8)	0.001
Peptic ulcer, n (%)	61 (0.4)	60 (0.4)	0.001
Peripheral vascular disease, n (%)	208 (1.2)	179 (1.0)	0.016
Pulmonary disease, n (%)	2588 (15.2)	2579 (15.1)	0.001
Renal disease, n (%)	484 (2.8)	430 (2.5)	0.020
White	12922 (75.7)	13027 (76.3)	0.014
Black or African American	2741 (16.1)	2668 (15.6)	0.012
Other race	583 (3.4)	577 (3.4)	0.002
Unknown race	522 (3.1)	512 (3.0)	0.003
Asian	162 (0.9)	152 (0.9)	0.006
American Indian or Alaska Native	91 (0.5)	90 (0.5)	0.001
Native Hawaiian or Other Pacific Islander	44 (0.3)	39 (0.2)	0.006
Opioid related disorders	152 (0.9)	133 (0.8)	0.012
Mean CCI	1.3	1.3	–

*Standardized mean difference (SMD) were used to assess baseline covariate balance; values <0.1 are considered indicative of adequate matching

## Results

A total of 110,287 patients were identified (Group 1: 17,098 and Group 2: 93,189). After propensity matching was performed, each group had 17,065 patients. After 90-days post-surgery, Group 1 (nicotine dependence) had higher odds of wound complications (OR: 1.599, 95%CI: 1.442-1.773), wound healing complications requiring intervention (OR: 1.581, 95%CI: 1.378-1.815), and seroma (OR: 1.512, 95%CI: 1.315-1.739). No differences were found among VTE rates (Table 2). Patients with nicotine dependence had higher odds of having an opioid prescription (OR: 1.515, 95%CI: 1.450-1.583) and had greater prescription events (2.50 ± 3.45 vs 2.07 ± 2.36; *p*<0.001) at 90-days postoperatively.

**Table 2:** Postoperative outcomes

Characteristics	Cohort 1	Cohort 2	*p*-value	OR (95% CI)
Wound complications	964 (5.6%)	616 (3.6%)	<0.001	1.599 (1.442–1.773)
Operative wound intervention	530 (3.1%)	339 (2.0%)	<0.001	1.581 (1.378–1.815)
Seroma	503 (2.9%)	336 (2.0%)	<0.001	1.512 (1.315–1.739)
Opioid fill	7428 (43.5%)	5754 (33.7%)	<0.001	1.515 (1.450–1.583)
Venous thromboembolism	169 (1.0%)	136 (0.8%)	0.058	1.245 (0.992–1.562)

## Discussion

In this large, multi-institutional analysis, patients with a recent history of nicotine dependence demonstrated significantly higher odds of wound complications, operative interventions, seroma formation, and prescription opioid fills within 90-days of body contouring surgery compared to matched nonusers. These findings are consistent with prior plastic surgery literature demonstrating increased wound morbidity among nicotine users in reconstructive settings and national database analyses.

Nicotine itself has been shown to impair wound healing through a variety of mechanisms. The most common etiology is via vasoconstriction resulting in decreased perfusion of blood to the tissue leading to ischemia [1, 2]. Smokers additionally have higher levels of carbon monoxide that compete with oxygen for hemoglobin binding, leading to poorly oxygenated blood flow [2]. Nicotine use has also been shown to attenuate the inflammatory healing response by reducing cell chemotactics and oxidative bactericidal mechanisms which may increase risk for infection [2]. Additionally, nicotine reduces the proliferation and migration of fibroblasts leading to decreased collagen synthesis and deposition required for wound healing [5]. These underlying mechanisms may reflect the higher rates of wound complications and subsequent wound healing operative interventions, highlighting both the medical and economic burden for patients.

Beyond wound-related outcomes, this study also identified higher odds for prescription opioids and frequency of prescribing events among nicotine users up to 90-days postoperatively. While prescriptions may not equate to consumption, this finding suggests that nicotine exposure may influence postoperative pain experiences and recovery trajectories. Chronic nicotine usage has been shown to alter pain pathways which may lead to hyperalgesia and increased pain scores postoperatively [4]. Heightened pain may decrease mobility and alter postoperative biomechanics potentially leading to increased wound morbidity. In the context of the opioid epidemic, higher postoperative opioid exposure and potential usage may elevate the risk for persistent use and misuse [4]. These findings highlight that nicotine cessation strategies represent an important component of perioperative optimization, with potential benefits extending beyond wound healing.

Given nicotine’s effects on microvascular perfusion, prior literature suggests that alternative forms of delivery, such as vaping or smokeless tobacco, may confer similar perioperative wound healing risks and warrants abstinence [6, 7]. The American College of Cardiology recommends a nicotine abstinence period of at least four weeks prior to surgery to reduce risk for wound healing complications [8]. Abstinence should be continued through the postoperative healing period, which may depend on surgery type [9]. Preoperative urine cotinine testing may be used to objectively confirm nicotine cessation, with detectability up to seven days after last use [10, 11]. The impact of marijuana usage remains less clearly defined in this population. However, existing surgical literature has demonstrated that increased wound healing complications and similar preoperative counseling should be performed by surgeons to mitigate risks. [12–14]

Limitations to this study are its retrospective design, reliance on coding accuracy, lack of granular patient data regarding nicotine administration, frequency, duration, cessation timing, and dosage. There is also lack of granular, patient-level opioid prescription data including tablet count and dosing, along with lack of admission status or readmission rates. These findings represent associations rather than causal relationships. Future research should examine the effects of marijuana usage on postoperative body contouring outcomes. In summary, nicotine dependence is associated with increased wound-related complications and opioid prescription fills following elective body contouring surgery. These results highlight the importance of preoperative risk stratification and reinforce nicotine cessation as a key component of perioperative optimization in aesthetic surgery.

## Supplementary Information

Below is the link to the electronic supplementary material.

Supplementary file1 (DOCX 13 kb)
