# Supplementary material for: The Impact of Nicotine Usage Across Body Contouring Surgery Outcomes and Opioid Usage
Source: Aesthetic Plast Surg. 2026 Jul 29;50(19):8007–10. doi: 10.1007/s00266-026-06033-5 (PMC13710416; doi:10.1007/s00266-026-06033-5)
Supplement: Supplementary file 1 [file 266_2026_6033_MOESM1_ESM.docx]

**SUPPLEMENTAL INFORMATION**

**Supplemental Table 1**: CPT Codes

| **Codes** | **Definition** |
| --- | --- |
| 15839 | Excision, excessive skin and subcutaneous tissue (includes lipectomy); other area |
| 15836 | Excision, excessive skin and subcutaneous tissue (includes lipectomy); arm |
| 15832 | Excision, excessive skin and subcutaneous tissue (includes lipectomy); thigh |
| 15834 | Excision, excessive skin and subcutaneous tissue (includes lipectomy); hip |
| 15835 | Excision, excessive skin and subcutaneous tissue (includes lipectomy); buttock |
| 15833 | Excision, excessive skin and subcutaneous tissue (includes lipectomy); leg |
| 15838 | Excision, excessive skin and subcutaneous tissue (includes lipectomy); submental fat pad |
| 15837 | Excision, excessive skin and subcutaneous tissue (includes lipectomy); forearm or hand |
| 15847 | Excision, excessive skin and subcutaneous tissue (includes lipectomy), abdomen (eg, abdominoplasty) (includes umbilical transposition and fascial plication) |
| 15877 | Suction assisted lipectomy; trunk |
| 15878 | Suction assisted lipectomy; upper extremity |
| 15879 | Suction assisted lipectomy; lower extremity |
| 19318 | Breast reduction |
| 19316 | Mastopexy |
